# Genome Wide Association Mapping of Seedling and Adult Plant Resistance to Barley Stripe Rust (*Puccinia striiformis* f. sp. *hordei*) in India

**DOI:** 10.3389/fpls.2018.00520

**Published:** 2018-04-24

**Authors:** Andrea Visioni, Sanjaya Gyawali, Rajan Selvakumar, Om P. Gangwar, Pradeep S. Shekhawat, Subhash C. Bhardwaj, Ayed M. Al-Abdallat, Zakaria Kehel, Ramesh P. S. Verma

**Affiliations:** ^1^Biodiversity and Integrated Gene Management, International Center for Agricultural Research in the Dry Areas, Rabat, Morocco; ^2^Department of Plant Science, University of Manitoba, Winnipeg, MB, Canada; ^3^Indian Institute of Wheat and Barley Research, Indian Council of Agricultural Research, Karnal, India; ^4^Rajasthan Agricultural Research Institute, Durgapura, Jaipur, India; ^5^Department of Horticulture and Crop Science, Faculty of Agriculture, The University of Jordan, Amman, Jordan

**Keywords:** *Puccinia striiformis* f. sp. hordei, GWAM, seedling stage, barley (*Hordeum vulgare* L.), adult plant resistance (APR), QTL

## Abstract

Barley stripe rust is caused by *Puccinia striiformis* f.sp. *hordei*, (Psh), occurs worldwide, and is a major disease in South Asia. The aim of this work was to identify and estimate effects of loci underlying quantitative resistance to rust at seedling and adult plant stages. HI-AM panel of 261 barley genotypes consisting of released cultivars from North and South America, Europe, Australia, advanced breeding lines, and local landraces from ICARDA barley program were screened at seedling and adult plant stages for resistance to Psh. Seedling resistance was evaluated with the five prevalent Psh races in India. Screening for the adult plant stage resistance was also performed in two different locations by inoculating with a mixture of the five races used for seedling screeing. The panel was genotyped using DaRT-Seq high-throughput genotyping platform. The genome-wide association mapping (GWAM) showed a total of 45 QTL located across the seven barley chromosomes for seedling resistance to the five races and 18 QTL for adult plant stage resistance. Common QTL for different races at seedling stage were found on all chromosomes except on chromosome 1H. Four common QTL associated with seedling and adult plant stage resistance were found on chromosomes 2, 5, and 6H. Moreover, one of the QTL located on the long arm of chromosome 5H showed stable effects across environments for adult plant stage resistance. Several QTL identified in this study were also reported before in bi-parental and association mapping populations studies validating current GWAM. However 15 new QTL were found at adult plant stage on all chromosomes except the 4H, explaining up to 36.79% of the variance. The promising QTL detected at both stages, once validated, can be used for MAS in Psh resistance breeding program globally.

## Introduction

Barley (*Hordeum vulgare* L.) is one of the most important cereal crops in the word and especially in the dry areas where often is the only crop that can be grown under extreme drought conditions (Ceccarelli, 1994; Li et al., 2006). On the other hand, barley managed by irrigation and high rainfall is common in South Asia and East Africa as well as other regions where rusts and foliar blights are important production constraints. The stripe rust caused by *Puccinia striiformis* f. sp. *hordei*, (Psh) is the major constraint in South Asia, East Africa, and Central and North America affecting both quantity and quality of barley produced (Luthra and Chopra, 1990; Roelfs and Huerta-Espino, 1994). Though, Psh can be effectively managed with fungicides, but the use of resistant varieties is considered the most sustainable option for both environmental and economic reasons. Incorporation of qualitative and quantitative resistance is important to obtain cultivars with durable resistance to stripe rust (St. Clair, 2010). Quantitative rust resistance is mediated by quantitative trait loci (QTL) conferring partial/non race specific resistance or “slow rusting” type of resistance. Many QTL reduce the disease severity by increasing rust incubation or latent periods (Mundt, 2014). Usually slow rusting genes have small or intermediate effects when present alone but a higher degree of durable resistance can be achieved by combining 4 or 5 such genes (Singh et al., 2000; Herrera-Foessel, 2011). Quantitative resistance is usually considered race-non-specific but as reported by Poland et al. (2009), the biological mechanisms underlying it is poorly understood and a wide variation of mechanisms is expected. Rust pathogens have intrinsic characteristic like wind-aided migration, ability to easily increase population size and to mutate and acquire new virulence to resistance genes. Due to these characteristics quantitative resistance is preferable to qualitative resistance for long lasting cultivation of new resistant cultivars. Furthermore, with more than 70 Psh races identified (Hovmøller, 2002) a better understanding of the genetic control of quantitative resistance is of crucial importance and breeding would be more effective if based on extensive knowledge of the resistance genes/QTL (Gutiérrez et al., 2015). A previuous study performed by Verma et al. (2018), using the same germplasm, identified accessions carrying already know resistance genes and Psh resistant genotypes to all the five races that may possess novel resistance genes. The current study was undertaken to identify QTL effective against Psh individual races at seedling stage and QTL for quantitative resistance in field globally and especially for South Asia where annual recurrence of stripe rust from the Himalayas is a big challenge.

## Materials and methods

### Plant material and yellow rust races

Two hundred sixty-one spring barley genotypes (172 two-row and 89 six-row types), including released cultivars, advanced breeding lines, and landraces were used in this study. The set is named as HI-AM (High Input Association Mapping) panel as mostly barley genotypes were from ICARDA breeding program targeted toward optimum management conditions. Out of the 261 genotypes, 124 were from ICARDA's barley breeding program (50 two-row and 74 six-row type), 32 from Europe (28 two-row and 4 six-row type), 34 North America (28 two-row and 6 six-row type), 67 from South America (62 two-row and 5 six-row type) and 4 from Australia (only two-row type). The seedling resistance test (SRT) for HI-AM was done against five prevalent Psh races in India, namely, Q (5S0), 24 (0S0-1), 57 (0S0), M (1S0), and G (4S0) under glasshouse at Regional Station, Indian Institute of Wheat and Barley Research (IIWBR), Shimla, India. Among these races, 57, 24, and G were old races while Q and M were identified and characterized more recently (Nayar et al., 1997; Prashar et al., 2007). The barley geneotypes were also screened at their Adult-plant stage (APS) at two locations using mixture of the five races received from Regional Station, IIWBR, Shimla, India.

### Screening for seedling resistance to five races of Psh

Seeds (5 seeds per hole per genotype) were sown in aluminum trays filled with a mixture of fine loam soil and farmyard manure. A total of 18 genotypes and a susceptible check “Bilara-2” were planted in each tray. Seedlings were inoculated 1 week when primary leaves were fully expanded. Inoculation was done using a glass atomizer with 100 mg of rust spores of each race suspended in 10 ml light grade mineral oil, Soltrol 170, (Chevron Phillips Chemicals Asia Pvt. Ltd., Singapore). Inoculated seedlings were misted with sterile water and placed for 48 h in dew chambers at 16 ± 2°C with >90% relative humidity and 12 h day/night cycle. Seedlings were then transferred to glasshouse and incubated at 16 ± 2°C with >70% relative humidity, illuminated at about 15,000 lux for 12 h. Leaves were treated with fine elemental sulfur to prevent powdery mildew infection, without affecting rust infections. Infection types (ITs), were recorded 16–18 days after inoculation following the modified methods of Nayar et al. (1997) and Stakman et al. (1962). Infection types 0 to 2 were considered resistant and 3 to 33^+^ as susceptible (Table 1A). Genotypes with resiant recation were tested again against the same races to confirm their reaction. The experiment was repeated once with resistant genotypes only to ascertain the consistency of the ITs.

Table 1Summary of Psh reaction types at seedling stage and adult plant stage.

**Table:** 

(A) Infection type^*^	**Number of genotypes**				
	Q (5S0)^**^	24 (0S0-1)^**^	^**^57 (0S0)^**^	M (1S0)^**^	G (4S0)^**^
‘0' ‘;-' ‘;'	39 (14.9)	141 (54.0)	73 (27.9)	162 (62.0)	121 (46.4)
‘1'	0 (0)	0 (0)	0 (0)	0 (0)	0 (0)
‘2' ‘2-' ‘2+'	2 (0.6)	2 (0.8)	9 (3.4)	0 (0)	3 (1.1)
**Resistant**	**43 (16.5%)**	**143 (54.8%)**	**82 (31.4%)**	**162 (62.0%)**	**124 (47.5%)**
‘3' ‘3-'	58 (22.2)	22 (8.4)	40 (16.8)	2 (0.8)	25 (9.5)
‘33+' ‘3+'	162 (62.1)	93 (35.6)	135 (51.7)	97(37.1)	111 (42.5)
**Susceptible**	**220 (84.3%)**	**115 (44.0%)**	**175 (67.0%)**	**99 (37.9%)**	**136 (52.1%)**

**Table:** 

(B) Rust severity^***^	**Number of genotypes**		
	**Durganpura 13**	**Durganpura 14**	**Karnal 14**
‘0, TR, TMR, TMS, TS'	48 (16.9)	34 (12.7)	193 (74.0)
‘ ≤ 20R, ≤ 10MR'	43 (16.4)	19 (7.2)	7 (2.7)
‘>10MR-40MR, 5MS'	31 (11.9)	20 (7.6)	1 (0.3)
**Resistant**	**122 (46.7%)**	**73 (27.9%)**	**201 (77.0%)**
‘5S-20S, >5MS'	96 (36.8)	130 (49.8)	52 (19.9)
‘>20S-60S'	18 (6.9)	27 (10.3)	4 (1.5)
‘>60S-100S'	25 (9.5)	30 (11.5)	1 (0.4)
**Susceptible**	**139 (53.2%)**	**187 (71.6%)**	**57 (21.8%)**

* *0; (naught fleck) no visible infection.;- (Fleck minus) slightly necrosis /micro-flecking visible.; (Fleck) no uredia but small hypersensitive flecks present. 1uredia minute, surrounded by distinct necrotic areas. 2 small to medium uredia surrounded by chlorotic or necrotic border. 3 uredia small to medium in size and chlorotic areas may be present. 3+ uredia large with or without chlorosis, sporulating profusely and forming rings. 33+ both 3 and 3+ pustules occur together*.

** *Race type of Puccinia striiformis Westend. f. sp. hordei Erikss*.

*** *R no uredia present. TR trace or minute uredia on leaves without sporulation. TMR trace or minute uredia on leaves with some sporulation. MR small uredia with slight sporulation. MR-MS small-to-medium-sized uredia with moderate sporulation. MS-S medium-sized uredia with moderate to heavy sporulation. S large uredia with abundant sporulation*.

### Screening for adult-plant stage resistance

All genotypes used in the seedling stage resistance screening were also evalauted for APS under artificial inoculation conditions at RARI, Durgapura (75° 47′ E, 26° 51′ N), Rajasthan, in crop seasons 2012–13 and 2013–14 and at ICAR-IIWBR, Karnal (76° 98′ E, 29° 69′ N),Haryana during 2013–2014, in India. The experiment was laid out in augmented design with yellow rust susceptible check, “Bilara-2,” after every 20 test genotypes in both locations and all seasons. Seeds were sown in 1-m rows with 25 cm row to row distance for each genotype in the first fortnight of November each year at Durgapura and Karnal. The infector rows of Bilara-2 were also sown perpendicular to the test material rows all around the blocks. In order to multiply the initial inoculum six rows of Bilara-2 (about 50 M long) were sown close in the same field about 15 days before the sowing of experimental materials. The five races Q (5S0), 24 (0S0-1), 57 (0S0), M (1S0), and G (4S0) received from Regional Station, IIWBR, Shimla were multiplied on this large plot of Bilara 2.

The infector rows were syringe inoculated at seedling stages (Zadoks GS 20) with the mixed inoculum of stripe rust races taken from early sown Bilara 2 plot, followed by repeated sprays of inoculum collected from infector rows as well as from early sown Bilara 2 plot on the test material. The field was given extra irrigations (sprinkler) to maintain an appropriate humid microclimate for better disease devlopment. Stripe rust severity was recorded at the early to late flowering stages (Zadoks GS 60-69) when maximum disease severity reached on the susceptible check rows, sown after every 20 rows of test material.

The modified Cobb's scale (Peterson et al., 1948) was used for classifying geneotypes to different reaction groups (Table 1B). Genotypes were classified as moderately resistant (up to 10 MR); moderately susceptible (5S to 20 MS); susceptible (>20MS-60S) and highly susceptible (>60S) based on stripe rust severity over two seasons and two locations.

### Genotyping with DaRT-Seq

Total genomic DNA extraction was performed on a single plant from each genotype from the HI-AM panel using a Biosprint 96 DNA Plant Kit (Qiagen, Hilden, Germany). DNA sample were processed for DaRT-Seq (Diversity Array Technology Pty Ltd, DaRT P/L) by a series of digestion/ligation reactions (Cruz et al., 2013; Ames et al., 2015). The system combines complexity reduction methods with next-generation sequencing platforms, targeting primarily genic regions (Carling et al., 2015). It produces two types of markers, SilicoDaRT markers characterized by presence/absence variation (PAVs) and classical SNPs present in the sequenced fragments (http://www.diversityarrays.com/dartapplication-dartseq-data-types). The PAV/SNP markers were subsequently aligned by using sequence information available at ftp://ftpmips.helmholtz-muenchen.de/plants/barley/public_data/anchoring. The Thresholds for minimum base id of 90% and E-value of 5^−10^ were imposed to declare positive matches against the available datasets of the physical map (ftp://ftpmips.helmholtz-muenchen.de/plants/barley/public_data/). Markers quality control of the initial dataset was conducted by removing heterozygous and monomorphic markers and markers with minor allele frequencies (MAF) < 5% and markers with missing data > 10%.

### Population structure and linkage disequilibrium

The genetic structure of the 261 genotypes of the HI-AM panel was investigated using 105 and 101 unlinked markers from the PAV set and from the SNP set, respectively, distributed across the 7 barley chromosomes. Markers subsets used for population structure assessment were obtained by selecting one marker every 10 cM within both PAVs and SNPs sets, in order to avoid to enclose linked markers in the subsets. Population structure was firstly determined using STRUCTURE version 2.3.4 (Pritchard et al., 2000). The admixture model option was run using a burn-in length of 10^10^ cycles, in order to minimize the effect of starting configuration, and a simulation of 10^6^ cycles was applied. Cluster values (k) from 2 to 10 were chosen and 5 independent runs for each k were chosen to obtain consistent results. Additionally, the *adegenet* package for R statistical software (The R Development core team) was used to confirm the number of sub-populations by the Bayesian Information Criterion (BIC). Finally, on the base of PCA results, genotypes were assigned to subgroups or considered admixed on the base of 80% membership criterion. A principal component analysis was also used to determine population structure and used as covariate in the subsequent GWAM study. Linkage disequilibrium (LD) was estimated with Tassel software V 5.2.32 (Bradbury et al., 2007) using a subset of 1,577 polymorphic markers with known position selected from the original SNP marker-set. Linkage disequilibrium was calculated, using the SNPs marker set, separately for locus pairs within the same chromosomes and between chromosomes. LD was estimated as the squared allele frequency correlations (*R*^2^) with only *p*-values ≤ 0.01 for each pair of loci considered as significant. The *nlstools* package for R Statistical Software (The R Development core team) was used to estimate the extent of LD by non-linear regression analysis on the basis of intrachromosomial r^2^ values (Hill and Weir, 1988; Remington et al., 2001).

### Genome wide association mapping

Disease severity scores at seedling and adult plant stages and the genotypic data were used to perform GWAM using Tassel V 5.2.32. GWAM was performed using both General Linear Model (GLM) and Mixed Linear Model (MLM) methods. The general equations for GLM and MLM are the followings: *y* = *Xa* + *e* and *y* = *Xa* + *Qb* + *Ku* + *e*, respectively. *X* denotes at the marker while *Q* is the Q-matrix obtained by STRUCTURE software and *K* is the kinship matrix (Q+K). The vector for phenotypes is indicated as *y* while *a* is the effect of marker fixed effects and *b* represent the vector of fixed effects, while *u* is an unknown vector of random additive genetic effects. Association analysis using the GLM model was performed incorporating as covariate population structure derived from PCA analysis (GLM+PCA model) or the Q-matrix (GLM+Q model) in order to avoid type I errors. The MLM model consider the familiar relatedness (the K model) and takes into consideration both population structure and familiar relatedness (Q+K and PCA+K models). The kinship matrix (K) was estimated using Tassel V 5.2.32 from the whole set of DaRT markers with unique position. For comparison we also conducted GWAM without any correction for population substructure. For all scans threshold of (−log_10_ *p* ≥ 3) was set for identifying significant marker-trait associations. Significant markers mapping within 5 cM of each other were considered as being linked to the same QTL and the marker with the highest *p*-value was chosen as representing the QTL. We firstly determined the critical *p*-value for the significance of marker-trait association using the false discovery rate (FDR). Since the FDR was found to be highly stringent and considering the stringency of the model used for accounting for population structure, in which most of the false positives were inherently controlled. Markers were declared significant at the *p* = 0.0001 [−*log(p)* = *3*] with the selected models according to a liberal approach (Chan et al., 2010).

### QTL alignment and candidate genes

To align detected QTL with those previously reported in different barley germplasm, we checked for the presence of common markers and/or positions in the barleymap database (http://floresta.eead.csic.es/barleymap/; Cantalapiedra et al., 2015), that allows to search the position of barley genetic markers on the Barley Physical Map (IBSC, 2012). The position of the marker representative of the QTL was compared with those of markers at QTL peaks reported in previous studies. Candidate genes search was done using the PGSB database^1^ that provides access to the barley gene annotation described by the IBSC (2012). The markers at the QTL peaks, were used to search, in the genomic region encompassing the QTL, for functional domains or genes functionally related with disease resistance mechanisms.

## Results

### Seedling and adult plant resistance

The statistical analysis of the phenotypic data collected has been already reported by Verma et al. (2018), while the reaction type of barley genotypes at SRT and APS are summarized in Table 1. The results showed that the genotypes of HI-AM panel were resistant to races 24 (54.8%) and M (62 %), followed by race G (47.5%), while for the races 57 (31.4%), and Q (16.5%). Races Q and 57 were the most virulent races where over 84 and 68% of genotypes were susceptible (Table 1A).

In the APS screening, 46.7 and 27.9% genotypes were resistant at Durgapura during 2013 and 2014, respectively and 77% resistant at Karnal during 2014 (Table 1B). Durgapura represents an optimal site for Psh due to climatic conditions (temperature and humidity) that favors Psh development while Karnal is characterized by the occurrence of severe winters that may limit the development and sporulation of the rust for the secondary spread. However, Karnal also represents the location in Haryana, which is also prone to stripe rust losses in the wider region. We observed a variation in the range of field reactions between *Dg13* and *Dg14*, as well as between Durgapura and Karnal reactions. As expected data collected in Durgapura indicated a wide range of reaction types when compared with data collected in Karnal. In both locations the susceptible check showed the maximum level of susceptibility (100S) indicating that the variations observed in reactions of the test genotypes are due to climatic factors and not because of escape or inoculum load.

### Marker statistics, population structure, and linkage disequilibrium

**Figure 1:**
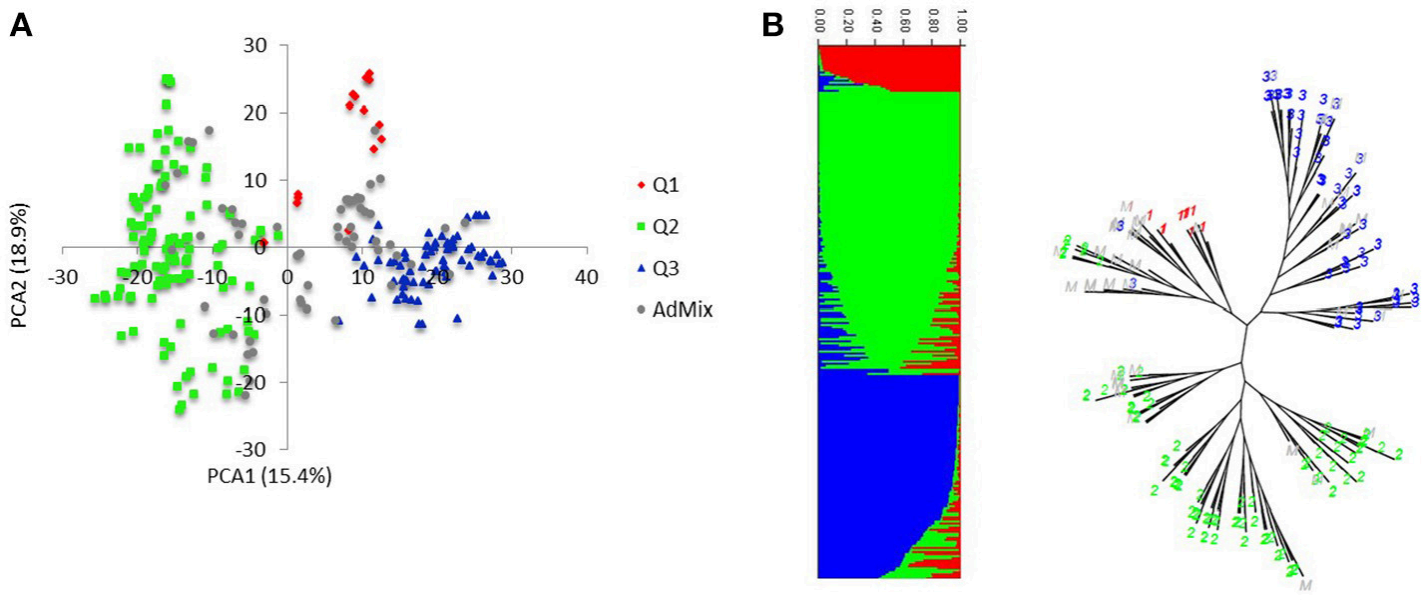
Population structure and linkage disequilibrium. **(A)** Principal component analysis of the HI-AM panel. **(B)** The proportion of the genome of each individual originating from each inferred sub-population, a total of 3 and, each color represent a single sub-population.

**Figure 2:**
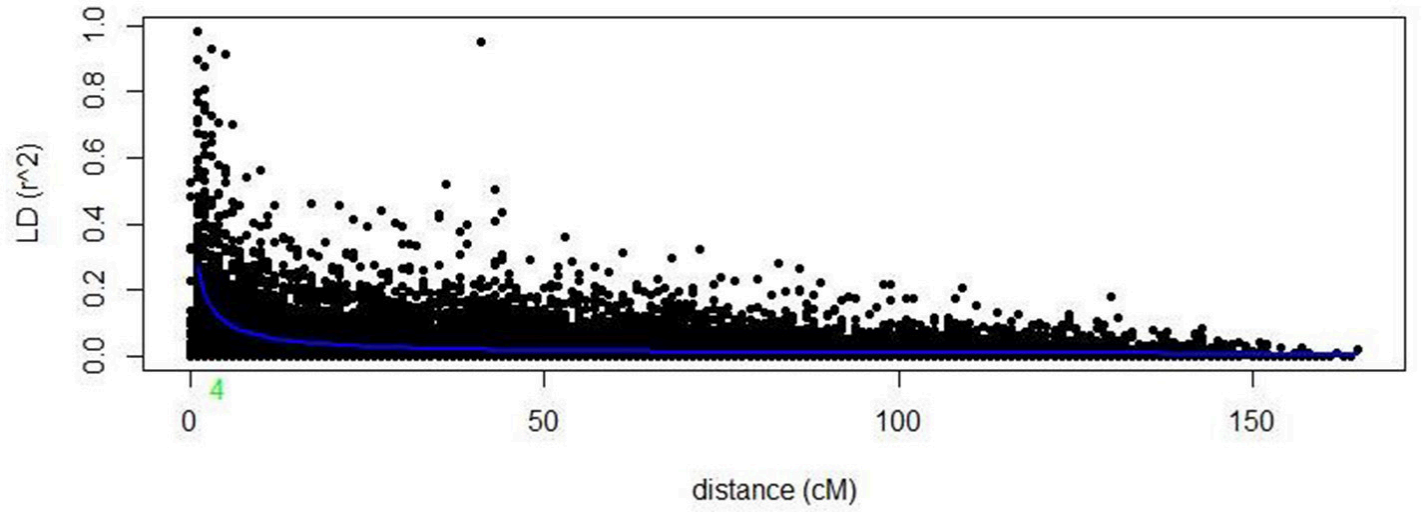
LD decay of the whole barley genome.

Markers selected after filtering steps have been subsequently used to estimate population structure, linkage disequilibrium (LD) and to perform GWAM for both SRT and APS. The final sets of markers comprise 13.182 PAV and 6.311 SNPs. Population structure analysis performed with Structure (Pritchard et al., 2000) showed three subpopulations (*k* = 3) on the base of the Δ*k* parameter and according to Evanno et al. (2005), the same number of k was also confirmed by the BIC estimation perfomed with R Statistical Software (The R Development core team). The population structure is shown in Figures 1A,B. The first subpopulation (Q1) is mainly composed by ICARDA germplasm (70%). The second group (Q2), located alone in the left side of the PCA chart (Figure 1A) shows the higher degree of diversity and the highest number of entries from South America (37%), followed by ICARDA germplasm (22%), North America (21%), Europe (13%), and Australia (3%). Q3 was again mainly composed by ICARDA germplasm (70%). The 6-row genotypes are spread across the three subgroups representing the 53% (Q1), 26% (Q2), and 45% (Q3) of the total number of genotypes for ech subpopulation, respectively. There were 153872 (24.4%) inter-chromosomal pairs of loci showing significant LD (*P* < 0.01), 3847 (2.5%) of which had *R*^2^ > 0.2. Of the intra-chromosomal locus pairs, 34227 (31.5%) had a significant LD of which 3022 (8.89%) had *R*^2^ > 0.2 Intra-chromosomal locus pairs have a higher mean *R*^2^ value (0.10) than inter-chromosomal locus pairs (0.02). The scatter plots of LD (*R*^2^) as a function of the inter-marker distance (cM) within the same chromosome for all genotypes indicated a clear LD decay at 4 cM (R^2^ = 0.18) with genetic distance (Figure 2).

### Genome wide association mapping

Using the PAVs marker set, the best model fitting was the MLM using Q+K model when analyzing SRT data; while PCA+K was the more suitable for APS data analyses. Using the SNP dataset the best fitting model for SRT and APS was the GLM model using PCA for accounting population structure and relatedness. The QQ plots for GWAM are available as Supplementary Materials (Figures S1–S4). The GWAM analyses at SRT identified 45 QTL located across the seven barley chromosomes (Table 2). The marker *R*^2^ ranged from 4.25% to 6.56% Table 2). The race specific QTL detected for SRT explained together the 41.77 % (Race Q), 50.1% (Race 24), 36.42% (Race 57), 53.0% (Race G), and 49.84% (Race M) of phenotypic variance, respectively. GWAM for APS showed 18 QTL using phenotypic data from two locations and during two seasons (Table 3). The marker *R*^2^ ranged from 4.54 to 8.11% for APS (Table 3). A QTL located on chromosome 5H was found consistantly stable across seasons and environments. Phenotypic variance explained by QTL detected in case of APS was 15.35% for *Dg13*, 36.79% for *Dg14* and 45.82% for *Kr14*. Among the QTL detected, 8 QTL were significant for two races (Table 4A). The QTL on chromosome 5H (cM 137.08), overlaps with a QTL for resistance at SRT for the race M. Furthermore, other 2 QTL detected for APS are located on chromosome 2H at the same position of QTL detected for resistance at SRT for race G (Table 4B). Similarly another QTL for APS located on chromosiome 6H overlaps with OTL for SRT for race Q.

**Table 2:** GWAM results for seedling resistance test to individual races.

**QTL**	**Marker**	**Chr**.	**Pos (cM)**	**–log_10_(p)**	**Marker R^2^ (%)**	**Effect**	**MAF (%)**
**RACE Q**							
*SRT_R0_1*	DaRT2415	2H	52.90	3.0248	4.43	1.75	19.92
*SRT_R0_2*	SNP1425	2H	140.72	3.2218	4.91	−0.50	10.92
*SRT_R0_3*	SNP1720	3H	51.63	3.1522	4.43	−1.77	31.13
*SRT_R0_4*	SNP2569	4H	61.12	4.0193	6.05	−0.21	27.53
*SRT_R0_5*	DaRT8922	5H	151.98	3.1837	4.93	−1.44	35.59
*SRT_R0_6*	SNP3974	6H	88.51	4.3196	6.41	1.73	26.88
*SRT_R0_7*	SNP4058	6H	113.24	3.6517	5.47	−2.71	7.72
*SRT_R0_8*	SNP4090	6H	118.77	3.3578	5.15	1.48	29.54
**RACE 24**							
*SRT_R24_1*	SNP50	1H	28.88	3.1535	4.25	−1.94	40.16
*SRT_R24_2*	DaRT4323	3H	7.01	3.2681	5.03	2.11	23.81
*SRT_R24_3*	DaRT4492	3H	14.94	3.1554	4.85	2.05	25.70
*SRT_R24_4*	SNP1579	3H	36.98	3.9825	5.51	3.25	16.60
*SRT_R24_5*	DaRT4767	3H	42.46	3.7706	6.07	2.56	25.41
*SRT_R24_6*	DaRT6458	4H	50.99	3.0864	4.73	−2.38	22.40
*SRT_R24_7*	DaRT7144	4H	111.33	3.1133	5.01	2.51	21.76
*SRT_R24_8*	DaRT7232	5H	3.02	3.1558	4.74	2.42	14.12
*SRT_R24_9*	DaRT8907	5H	151.88	3.0918	4.79	2.01	26.03
*SRT_R24_10*	DaRT11535	7H	24.06	3.3674	5.12	2.26	22.53
**RACE 57**							
*SRT_R57_1*	DaRT1073	1H	93.06	3.7433	6.09	2.26	18.30
*SRT_R57_2*	DaRT3036	2H	94.55	3.3503	5.05	1.98	21.18
*SRT_R57_3*	SNP1637	3H	45.22	3.1389	4.52	2.30	21.83
*SRT_R57_4*	DaRT5092	3H	59.63	3.1588	4.64	1.87	25.39
*SRT_R57_5*	SNP3013	5H	55.62	3.0613	4.28	2.70	11.20
*SRT_R57_6*	DaRT11344	7H	15.37	3.2730	5.29	1.99	29.66
*SRT_R57_7*	DaRT11479	7H	23.02	3.9349	6.56	2.04	42.37
**RACE G**							
*SRT_RG_1*	DaRT1886	2H	8.85	3.3314	5.18	−1.84	47.56
*SRT_RG_2*	SNP1434	2H	140.79	3.0564	4.33	2.19	36.36
*SRT_RG_3*	DaRT3982	2H	146.72	3.3517	5.04	2.33	19.29
*SRT_RG_4*	DaRT6779	4H	68.98	3.4257	5.57	2.19	22.46
*SRT_RG_5*	DaRT7132	4H	110.20	3.0167	4.59	2.10	20.33
*SRT_RG_6*	SNP2849	5H	32.88	3.0373	4.56	−2.65	13.39
*SRT_RG_7*	DaRT8539	5H	129.65	3.8616	5.88	2.03	30.35
*SRT_RG_8*	DaRT10087	6H	53.75	3.4969	5.27	3.53	6.64
*SRT_RG_9*	DaRT11815	7H	46.39	3.8967	6.28	2.04	46.72
*SRT_RG_10*	DaRT12391	7H	97.10	4.0060	6.29	2.84	13.15
**RACE M**							
*SRT_RM_1*	DaRT872	1H	64.02	3.0686	4.55	−3.54	6.61
*SRT_RM_2*	SNP1972	3H	98.23	3.0077	4.25	2.08	20.24
*SRT_RM_3*	DaRT6490	4H	51.42	4.0400	6.76	2.42	46.25
*SRT_RM_4*	DaRT6935	4H	91.50	3.2716	5.00	2.29	21.43
*SRT_RM_5*	DaRT8426	5H	120.35	3.0441	4.63	1.77	41.06
*SRT_RM_6*	DaRT8567	5H	133.69	3.0979	4.79	1.82	43.50
*SRT_RM_7*	SNP3724	6H	51.77	3.1876	4.41	2.72	13.46
*SRT_RM_8*	DaRT10299	6H	68.20	3.0433	4.85	1.77	40.25
*SRT_RM_9*	DaRT12705	7H	118.48	3.1742	4.89	2.52	15.48
*SRT_RM_10*	DaRT13019	7H	132.65	3.4122	5.71	2.15	27.12

**Table 3:** GWAM results for Psh resistance at adult plant stage.

**QTL**	**Marker**	**Chr**.	**Pos (cM)**	**−log_10_(p)**	**Marker R2 (%)**	**Effect**	**MAF (%)**
**DURGAPURA 2013**							
APS_Dg13_1^*^	DaRT493	1H	40.5	3.0075	4.69	18.70	35.74
APS_Dg13_2^*^	DaRT3562	2H	129.09	3.0026	4.92	23.11	8.33
*APS_Dg13_3*	DaRT8667	5H	137.08	3.7524	5.74	−25.80	9.77
**DURGAPURA 2014**							
APS_Dg14_1^*^	DaRT568	1H	47.52	4.0543	6.73	21.47	24.17
*APS_Dg14_2*	SNP632	2H	8.85	3.1469	4.79	−16.27	26.56
APS_Dg14_3^*^	SNP733	2H	40.08	3.5651	5.34	−15.79	33.06
APS_Dg14_4^*^	SNP1886	3H	78.21	3.0723	4.54	−13.43	35.77
*APS_Dg14_5*	DaRT8661	5H	137.08	3.3738	5.07	−19.21	19.84
*APS_Dg14_6*	DaRT10854	6H	119.12	3.1978	4.94	13.18	15.98
APS_Dg14_7^*^	SNP4572	7H	89.63	3.7015	5.38	−22.30	12.06
**KARNAL 2014**							
APS_Kr14_1^*^	SNP447	1H	103.82	3.5960	5.36	−9.32	16.19
APS_Kr14_2^*^	SNP476	1H	109.37	3.3382	4.84	−8.98	16.21
APS_Kr14_3^*^	DaRT2151	2H	26.20	3.0050	5.36	8.23	42.13
APS_Kr14_4^*^	DaRT2743	2H	64.83	3.2879	5.94	−7.89	22.00
APS_Kr14_5^*^	DaRT2798	2H	70.53	4.3200	8.11	−13.42	8.96
*APS_Kr14_6*	DaRT4067	2H	149.26	3.2942	5.73	−13.77	5.77
*APS_Kr14_7*	DaRT8668	5H	137.08	3.0786	5.30	−11.44	8.98
APS_Kr14_8^*^	SNP3509	5H	159.51	3.4386	5.18	−9.31	17.96

* *Putative Adult Plant Resistance (ADP) QTL*.

**Table 4:** Summary of co-located QTL at seedling adult plant stages.

**(A) SEEDLING STAGE**								
**QTL**	**Race**	**Marker**	**Chr**.	**Pos (cM)**	**–log_10_(p)**	**Marker R2 (%)**	**Effect**	**MAF (%)**
Sdl_R0_2	RaceQ	SNP1425	2H	140.72	3.2218	4.91	−0.50	10.92
Sdl_RG_2	RaceG	SNP1434	2H	140.79	3.0564	4.33	2.19	36.36
Sdl_R24_5	Race24	DaRT4767	3H	42.46	3.7706	6.07	2.56	25.41
Sdl_R57_3	Race57	SNP1637	3H	45.22	3.1389	4.52	2.30	21.83
Sdl_R24_6	Race24	DaRT6458	4H	50.99	3.0864	4.73	−2.38	22.40
Sdl_RM_3	RaceM	DaRT6490	4H	51.42	4.0400	6.76	2.42	46.25
Sdl_RG_5	RaceG	DaRT7132	4H	110.20	3.0167	4.59	2.10	20.33
Sdl_R24_7	Race24	DaRT7144	4H	111.33	3.1133	5.01	2.51	21.76
Sdl_RG_7	RaceG	DaRT8539	5H	129.65	3.8616	5.88	2.03	30.35
Sdl_RM_6	RaceM	DaRT8567	5H	133.69	3.0979	4.79	1.82	43.50
Sdl_R24_9	Race24	DaRT8907	5H	151.88	3.0918	4.79	2.01	26.03
Sdl_R0_5	RaceQ	DaRT8922	5H	151.98	3.1837	4.93	−1.44	35.59
Sdl_RM_7	RaceM	SNP3724	6H	51.77	3.1876	4.41	2.72	13.46
Sdl_RG_8	RaceG	DaRT10087	6H	53.75	3.4969	5.27	3.53	6.64
Sdl_R24_10	Race57	DaRT11479	7H	23.02	3.9349	6.56	2.04	42.37
Sdl_R57_7	Race24	DaRT11535	7H	24.06	3.3674	5.12	2.26	22.53
**(B) SEEDLING AND ADULT PLANT**								
**QTL**	**Trial/Race**	**Marker**	**Chr**	**Pos (cM)**	**–log**_10_**(p)**	**Marker R2 (%)**	**Effect**	**MAF (%)**
Sdl_RM_6	RaceM	DaRT8567	5H	133.69	3.0979	4.79	1.82	43.50
Ap_Dg13_3	DG13	DaRT8667	5H	137.08	3.7524	5.74	−25.80	9.77
Ap_Dg14_5	DG14	DaRT8661	5H	137.08	3.3738	5.07	−19.21	19.84
Ap_Kr13_7	KR13	DaRT8668	5H	137.08	3.0786	5.30	−11.44	8.98
Sdl_RG_1	RaceG	DaRT1886	2H	8.85	3.3314	5.18	−1.84	47.56
Ap_Dg14_2	DG14	SNP632	2H	8.85	3.1469	4.79	−16.27	26.56
Sdl_R0_7	RaceQ	SNP4090	6H	118.77	3.3578	5.15	1.48	29.54
Ap_Dg14_6	DG14	DaRT10854	6H	119.12	3.1978	4.94	13.18	15.98
Sdl_RG_3	RaceG	DaRT3982	2H	146.72	3.3517	5.04	2.33	19.29
Ap_Kr13_6	KR13	DaRT4067	2H	149.26	3.2942	5.73	−13.77	5.77

### Candidate genes for resistance to Psh

Out of the 45 QTL identified for resistance at SRT, 15 were coincident with prior reports Those QTL were reported from different barley germplasm and different Psh races (Thomas et al., 1995; Toojinda et al., 2000; Vales et al., 2005; Rao et al., 2007; Verhoeven et al., 2011; Gutiérrez et al., 2015; Dracatos et al., 2016; Esvelt Klos et al., 2016; Belcher et al., 2018). For Psh resistance at the APS stage using different germplasm and races, only 3 out of the 18 QTL detected, shown in Table 5, are coincident with previous reports (Vales et al., 2005; Rao et al., 2007; Verhoeven et al., 2011; Gutiérrez et al., 2015; Dracatos et al., 2016; Belcher et al., 2018).

**Table 5:** QTL aligned and candidate genes identified for seedling and adult plant stages.

**QTL**	**Chr**.	**Pos (cM)**	**Gene Identifier**	**Description**	Known co-segregating loci^**^
**(a) SEEDLING STAGE**					
**Race Q**					
*SRT_R0_1*	2H	52.90	MLOC_8615.2	Glucan endo-1,3-beta-glucosidase 4	11_10796 [(Toojinda et al., 2000) (APS); (Vales et al., 2005) (APS); (Gutiérrez et al., 2015) (APS)]
*SRT_R0_4*	4H	61.12	AK356118	Glucan endo-1,3-beta-glucosidase 4	–
*SRT_R0_5*	5H	151.98	MLOC_6270.1	NBS-LRR disease resistance protein family-1	SCRI_RS_2824 [(Verhoeven et al., 2011) (APS); (Gutiérrez et al., 2015) (APS); (Belcher et al., 2018) (APS)]
*SRT_R0_7*	6H	113.24	MLOC_67477.1	Disease resistance protein (CC-NBS-LRR class) family	–
*SRT_R0_8*	6H	118.77	AK370472	Disease resistance protein	
**RACE 24**					
*SRT_R24_1*	1H	28.88	MLOC_74415.1	Lr21	–
*SRT_R24_2*	3H	7.01	MLOC_62179.1	NBS-LRR disease resistance protein, putative	–
*SRT_R24_3*	3H	14.94	MLOC_75090.1	Endo-1,4-beta-xylanase check	–
*SRT_R24_4*	3H	36.98	MLOC_56904.1	NBS-LRR disease resistance protein homolog	–
*SRT_R24_5*	3H	42.46	–	–	SCRI_RS_154973 [(Rao et al., 2007) (APS); (Belcher et al., 2018) (APS)]
*SRT_R24_6*	4H	50.99	–	–	11_20853 [(Vales et al., 2005) (APS); (Gutiérrez et al., 2015) (APS); (Esvelt Klos et al., 2016) (SDL)]
*SRT_R24_7*	4H	111.33	AK365216	Disease resistance-responsive (dirigent-like protein) family protein	12_31138 [(Verhoeven et al., 2011) (APS); (Gutiérrez et al., 2015) (APS); (Belcher et al., 2018) (APS)]
*SRT_R24_8*	5H	3.02	MLOC_67608.3	NBS-LRR disease resistance protein, putative	-
*SRT_R24_9*	5H	151.88	MLOC_6270.1	NBS-LRR disease resistance protein family-1	SCRI_RS_2824 [(Verhoeven et al., 2011) (APS); (Gutiérrez et al., 2015) (APS); (Belcher et al., 2018) (APS)]
*SRT_R24_10*	7H	24.06	MLOC_67182.3	Cc-nbs-lrr resistance protein	–
**RACE 57**					
*SRT_R57_2*	2H	94.55	–	–	3259480|F|0 [(Dracatos et al., 2016) (SDL)
*SRT_R57_3*	3H	45.22	–	–	SCRI_RS_154973 [(Rao et al., 2007) (APS)]
*SRT_R57_4*	3H	59.63	MLOC_51359.1	NBS-LRR-like protein	–
*SRT_R57_6*	7H	15.37	MLOC_5217.3	Disease resistance protein (CC-NBS-LRR)	–
*SRT_R57_7*	7H	23.02	MLOC_67182.3	Cc-nbs-lrr resistance protein	–
**RACE G** –					
*SRT_RG_1*	2H	8.85	MLOC_73747.1	Cc-nbs-lrr resistance protein	–
*SRT_RG_3*	2H	146.72	MLOC_58526.2	Disease resistance protein (CC-NBS-LRR)	–
*SRT_RG_4*	4H	68.98	MLOC_74055.1	NAC domain protein	–
*SRT_RG_5*	4H	110.20	AK365216	Disease resistance-responsive (dirigent-like protein) family protein	12_31138 [(Verhoeven et al., 2011) (APS) (Gutiérrez et al., 2015) (APS); (Belcher et al., 2018) (APS)]
*SRT_RG_7*	5H	129.65	AK356729	Glucan endo-1,3-beta-glucosidase 3	11_11532 [(Vales et al., 2005) (APS); (Gutiérrez et al., 2015) (APS)]
*SRT_RG_8*	6H	53.75	MLOC_13229.1	Disease Resistance Protein	–
*SRT_RG_10*	7H	97.10	MLOC_16158.3	NB-ARC domain-containing disease resistance protein	–
**RACE M** –					
*SRT_RM_1*	1H	64.02	MLOC_59979.1	NBS-LRR disease resistance protein homolog	–
*SRT_RM_2*	3H	98.23	–	–	11_21212 [(Vales et al., 2005) (APS); (Gutiérrez et al., 2015) (APS)]
*SRT_RM_3*	4H	51.42	–	–	11_20853 [(Vales et al., 2005) (APS); (Gutiérrez et al., 2015) (APS); (Esvelt Klos et al., 2016) (SDL)]
*SRT_RM_5*	5H	120.35	MLOC_30580.2	NBS-LRR disease resistance protein	–
*SRT_RM_6*	5H	133.69	MLOC_10360.2	NBS-LRR disease resistance protein, putative	11_11532 [(Vales et al., 2005) (APS); (Gutiérrez et al., 2015) (APS)]
*SRT_RM_10*	7H	132.65	MLOC_38424.1	NBS-LRR disease resistance protein	11_10843 [(Thomas et al., 1995) (APS); (Gutiérrez et al., 2015) (APS); (Dracatos et al., 2016) (SDL)]
**(a) ADULT PLANT STAGE**					
**DURGAPURA 2013**					
APS_Dg13_1^*^	1H	40.5	MLOC_11791.2	Disease Resistance Protein	12_30817 [(Verhoeven et al., 2011) (APS); (Belcher et al., 2018) (APS)]
*APS_Dg13_3*	5H	137.08	MLOC_63574.2	Glucan endo-1,3-beta-glucosidase 5	–
**DURGAPURA 2014**					
APS_Dg14_1^*^	1H	47.52	MLOC_4500.2	NBS-LRR disease resistance protein homolog	–
*APS_Dg14_2*	2H	8.85	MLOC_78849.2	Disease resistance protein (TIR-NBS-LRR class)	–
*APS_Dg14_5*	5H	137.08	MLOC_63574.2	Glucan endo-1,3-beta-glucosidase 5	–
*APS_Dg14_6*	6H	119.12	MLOC_43055.1	Disease Resistance Protein	–
**KARNAL 2014**					
APS_Kr14_1^*^	1H	103.82	MLOC_54911.1		3263737|F|0 [(Dracatos et al., 2016) (SDL)]
APS_Kr14_4^*^	2H	64.83	MLOC_34376.1	Endo-1,4-b-D-glucanase	3258146|F|0 [(Dracatos et al., 2016) (SDL)]
*APS_Kr14_6*	2H	149.26	MLOC_19010.2	TIR-NBS-LRR class disease resistance protein	–
APS_Kr14_7^*^	5H	137.08	MLOC_63574.2	Glucan endo-1,3-beta-glucosidase 5	–
APS_Kr14_8^*^	5H	159.51	MLOC_58845.1	NBS-LRR disease resistance protein family-1	–

* Putative Adult Plant Resistance QTL;

** *SRT: Seedling Resistance Test; APS: Adult Plant Stage*.

Candidate genes (CG) were found for both QTL at the SRT and APS (Table 5). QTL detected at both SRT and APS are located in genomic regions enriched in genes or functional domains that according to their annotations can be considered indicative of common R gene products like: nucleotide binding site (NBS), leucine rich repeat (LLR) and disease resistance protein, genes involved in b-glucan biosynthesis. We identified 27 (CG) for Psh resistance at SRT and 10 for APS. As expected most of the CGs belongs to the NBS-LLR disease resistance protein family, others are classified as generic disease resistance proteins and also as genes involved in β-glucans biosynthesis. We also find NAC protein overlapping with the QTL *SRT _RG_4* on chromosome 4H and *Lr21* for the QTL *SRT _R24_1* located on the chromosome 1H.

## Discussion

Barley stripe rust specialization and race structure are poorly defined as compared with wheat rusts and only few studies on the genetic control of the resistance are available in barley. The lack of barley genetic stocks for resistance represent one of the main limiting factors in the identification of genetic determinats of Psh resistance (Dracatos et al., 2016). In order to identify genomic regions controlling resistance to five prevalent races of Psh in India at both SRT and APS stages, the study was taken up on the barley genotypes from much diverse sources. The SRT under artificial inoculation for the five races of the HI-AM panel showed that the most virulent races were Q and 57, respectively. For APS resistance, we observed variation in rust severity in the two seasons in Durgapura (*Dg13* vs. *Dg14*), however, the range of field reaction was higher at Durgapura as compared to Karnal (*Kr14*). Durgapura is an optimal location for rust screening due to relatively less severe winter favoring faster stripe rust development (Verma et al., 2018), while, Karnal station with severe winters sometimes experience delayed strip rust development with less secondary spread. However, the susceptible check Bilara-2 showed high severy with susceptible reaction at both locations indicating that there is no escape and it was possible to select resistant genotypes at both locations. Beside climate variation, others factors also contribute to the unmanageable variation in infection response in field trials. Inoculum composition, sequential infection, and plant phenology are others main factors that can modify plant response to Psh and others pathogens (Hickey et al., 2011; Gutiérrez et al., 2015). Therefore testing genotypes in different environments is important to confirm the resistance of barley genotypes to Psh.

As mentioned before the HI-AM comprises 261 spring genotypes from different breeding programs in different continents and landraces from germplasm collections. Ear type and origin of germplasm are often the main determinants of population subgrouping but in our case seems that both traits do not have any effect on population structure. The absence of subgrouping based on ear type may be due to the fact that the genotypes from breeding programs represent outcome of 2 × 6-row hybridization being frequently used for germplasm improvement. Furthermore out of 89 six-row genotypes present in the panel, 73 were bred and selected at ICARDA, where crosses between 2 and 6-row genotypes are routinary. In the case of germplasm origin, the absence of subgrouping is most probably due to the extensive germplasm exchange between ICARDA and breeders in North and South American. The distribution of genotypes across the three different subgroups seems to be due to the breeding history of individuals, and further analysis based on pedigrees might be helpful to understand relationship of genotypes belonging to different groups.

Normally seedling resistance is considered qualitative and based on gene for gene interaction between host and pathogen while quantitative resistance has been defined as a non-race specific resistance expressed only in adult plants (Milus and Line, 1986; Richardson et al., 2006). APR is generally best expressed at adult phase and usually involve additive and/or epistatic effects of multiple genes that confers a durable partial resistance (Hickey et al., 2011). Those QTL may represent an interesting source of quantitative resistance and, if validated they can be introgressesed in breeding materials, through MAS, to combine both qualitative and quantitative resistance. Qualitative resistance mechanisms have been extensively studied in terms of genomic location and specificity (Giese et al., 1993; Thomas et al., 1995; Graner and Tekauz, 1996) while mechanisms underlying quantitative resistance still to be clarified. As reviewed by Richardson et al. (2006) quantitative resistance may be controlled by uncharacterized classes of R genes or by alternative alleles at qualitative loci. For instance, Castro et al. (2002) reported two QTL located on chromosomes 4 and 5H conferring resistance to three Psh isolates (Psh-1, Psh-13, and Psh-14) both at SRT and APS, in the Shyri × Galena double haploid population. Those QTL are located in the same genomic regions where several authors reported multiple qualitative and quantitative resistance genes conferring resistance to different pathogens (von Wettstein-Knowles, 1992; Thomas et al., 1995; Qi et al., 1998; Hayes et al., 2000). We detected several race specific QTL at SRT stage that were already reported as QTL for resistance at both SRT and APS in prior reports. The fact that most of them were detected at APS and using different races support the hypothesis that both qualitative and quantitative resistance genes may be located at the same loci.

Interestingly the phenotypic variance explained by the QTL detected for the two most virulent races at SRT stage was the lowest (41.77% for race Q and 36.42% for race 57, respectively) when comparated with less virulent races. We detected 18 QTL for APS using a mixture of the five races, out of which only 3 were reported before. *APS_Dg14_1* detected at APS (Verhoeven et al., 2011), while *APS_Kr14_1* and *APS_Kr_14_4* were detected by Dracatos et al. (2016) at SRT. Furthermore, we found that eight of the QTL detected for SRT were significant for two different races, furthermore QTL *SRT _R0_2/SRT _RG_2, SRT _R24_6/SRT _RM_3* and *SRT _R24_9/SRT _R0_5* shows opposite effects within races. We also found that 13 out of 18 QTL detected at APS were not coincident with those detected at SRT, therefore they can be considered QTL for Adult Plant Resistance (APR). Among the QTL detected the most interesting are located on the long arm of chromosome 5H at cM 137.08 (*APS_Dg13_3, APS_Dg14_5, and APS_Kr_7)*, near the telomeric region, it was significant across environments and represents the best candidate for validation detected at APS stage. Commons QTL for SRT and APS resistance are located on chromosomes 2HS, 2HL, 5H, and 6HL respectively. It is noteworthy to mention that the QTL for APS on chromosome 5H at 137.08 cM is overlapping with QTL *SRT_RM_6*. The QTL *SRT_RM_6* is located 3.39 cM (133.69 cM) from the QTL for APS and is position is coincident with another QTL previously reported for APS by Vales et al. (2005) and by Gutiérrez et al. (2015) located at 129.44 cM in the IBSC 2012^2^ barley genetic map.

Again this could be explained by presence of clusters of R genes where qualitative genes may regulate the response in SRT, while quantitative genes control the non-race specific response at APS. In case of the overlapping race specific QTL, the opposite effects detected between different races may be due to different race specific resistance genes at the same loci or to the same resistance QTL/gene that responds in a race specific way. Since, as reported by Park (2008) the use of both SRT and APR is desirable for increasing durability of rust resistance in cereals those QTL also represent a valuable source of resistance to Psh. Furthermore, is noteworthy that the most part of QTL detected at APS stage shows negative effect on rust pathogenesis and if validated, they can be used in MAS in stripe rust resistance breeding in barley, especially in India and South Asia.

For several of QTL detected at both SRT and APS we were able to identify CGs; preference was given to genes predicted to be members of the most common family of R genes and other genes predicted to be relevant to stripe rust pathogenesis. QTL genomic region encompass genes annotated for functions or for domains related to disease resistance thus these genes can be considered putative candidate genes for the corresponding QTL. Those genes are part of most represented resistance gene family that encodes cytoplasmatic proteins with nucleotide-binding sites and several leucine rich repeats (Halterman et al., 2001). Furthermore we also found several candidate genes annotated as part of the in β-glucans biosynthesis pathway. β-glucans are component of cell wall and glucanases have a role in early plant response to fungal pathogens. In fact glucanases are secreted by cell walls that are directed toward degradation of fungal walls (Thomma, 2003; Veronese et al., 2003; Vorwerk et al., 2004). The CG associated with race specific QTL *SRT_RG_4* was a protein with a NAC domain; proteins encoded by NAC gene family constitute a large family of specific transcription factors, involved in both abiotic and biotic stress response (Al Abdallat et al., 2014; McGrann et al., 2015). For instance, the wheat transcription factor *TaNAC4* expression is induced in leaves by the infection of stripe rust (Xia et al., 2010). Many other genes implicated in plant defense response process may be co-located in the same regions. For instance Esvelt Klos et al. (2016) reported several CGs related with cellular reactive oxygen species that are known to play an important role in plant defense mechanisms. Different hypothesis, based on experimental evidences, have been postulated on the mechanisms underlying the quantitative disease resistance and further studies are required to decipher how this mechanisms confers resistance to disease like Psh.

## Conclusion

Expanding the catalog of mapped QTL for stripe rust resistance and its validation represent an important step toward the application of MAS for the introgression and pyramiding of resistance genes in new barley cultivars. In this work, novel QTL for Psh resistance at SRT and adult plant stages were identified which could be helpful in dissection the resistance mechanism to this pathogen. New QTL need to be validated for their diversity, effectiveness in different genetic background and with more races of Psh existing in other regions of the world to ensure their use for introgression in barley germplasm or for MAS globally.

## Author contributions

RV, SG, and AV: conceived and coordinated the study; AV, SG, AA-A, and ZK: performed statistical and bioinformatic analysis; RS, OG, PS, and SB: collected the phenotypic data; AV, RV, SG, and AA-A: reviewed and contributed to draft the manuscript.

### Conflict of interest statement

The authors declare that the research was conducted in the absence of any commercial or financial relationships that could be construed as a potential conflict of interest.

## Footnotes

^1^Plant Genome System Biology (PGSB). Available online at: http://pgsb.helmholtz-muenchen.de/plant/index.jsp

^2^International Barley Genome Sequencing Consortium (IBSC, 2012) Available online at: http://barleygenome.org
