# Supplementary material for: Genome Wide Association Mapping of Seedling and Adult Plant Resistance to Barley Stripe Rust (Puccinia striiformis f. sp. hordei) in India
Source: Front Plant Sci. 2018 Apr 24;9:520. doi: 10.3389/fpls.2018.00520 (PMC5928535; doi:10.3389/fpls.2018.00520)

**Supplementary material:**

Sup. Figure 1. QQ plots for SRT marker-trait association analysis using the PAVs markers set and the following four models: GLM + Q, GLM +PCA, MLM (K+Q Model) and MLM (PCA+K Model). The black line is the expected line under the null distribution.


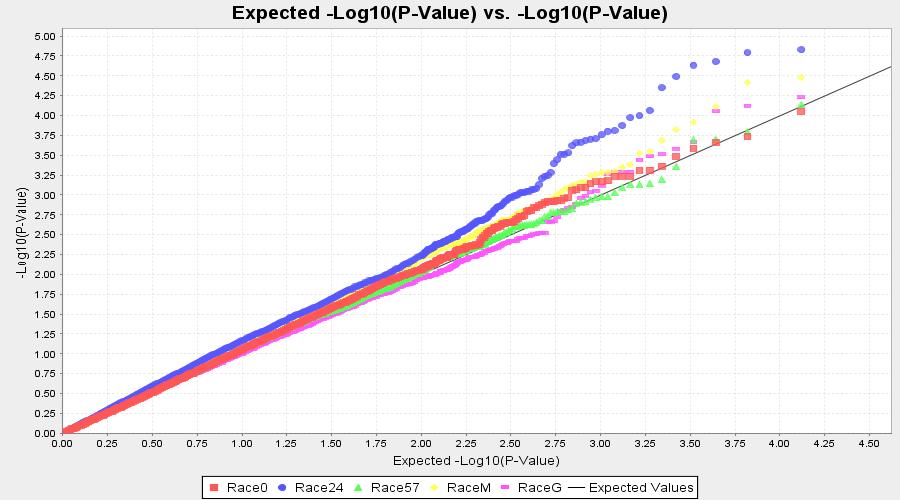


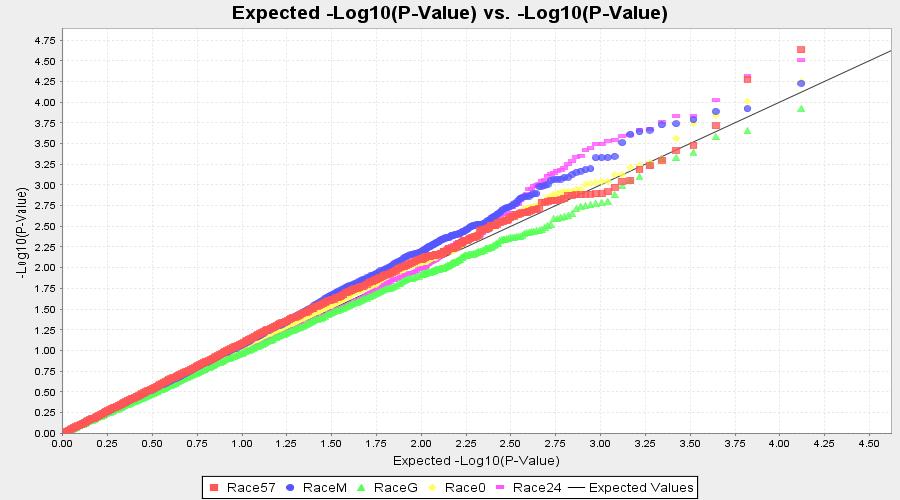


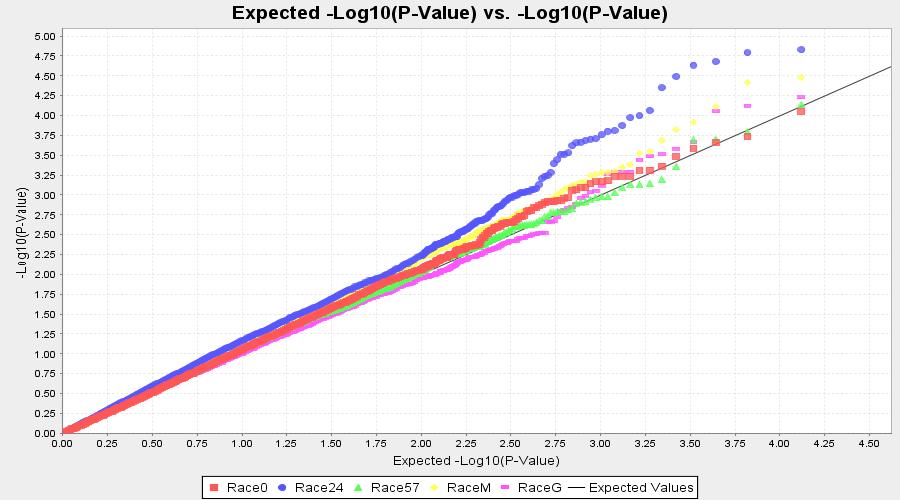


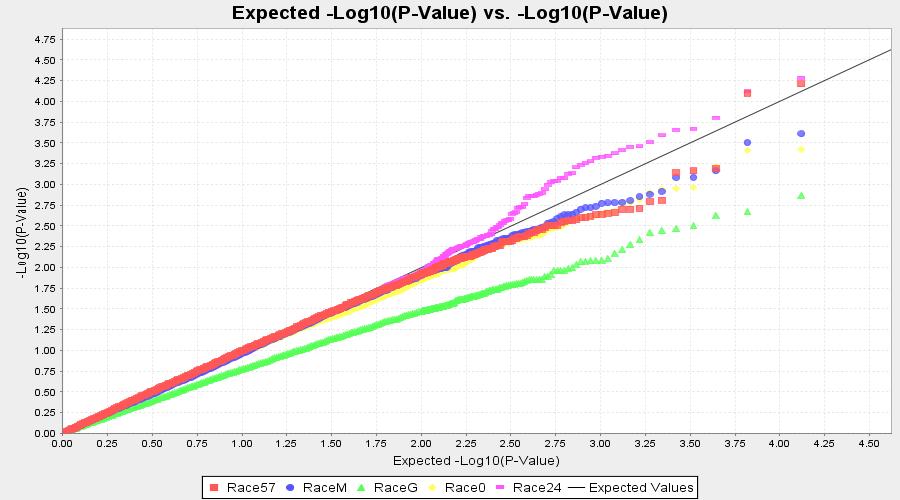


Sup. Figure 2. QQ plots APS marker-trait association analysis using the PAVs markers set and the following four models: GLM + Q, GLM +PCA, MLM (K+Q Model) and MLM (PCA+K Model). The black line is the expected line under the null distribution.


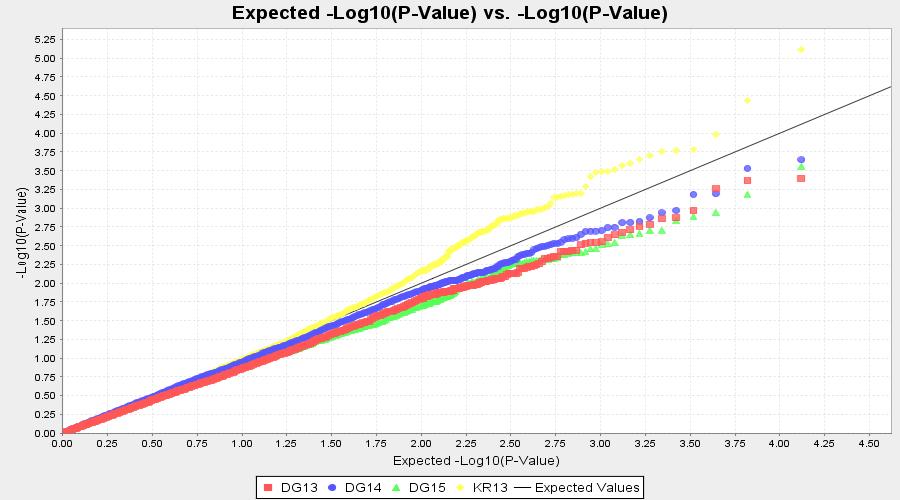


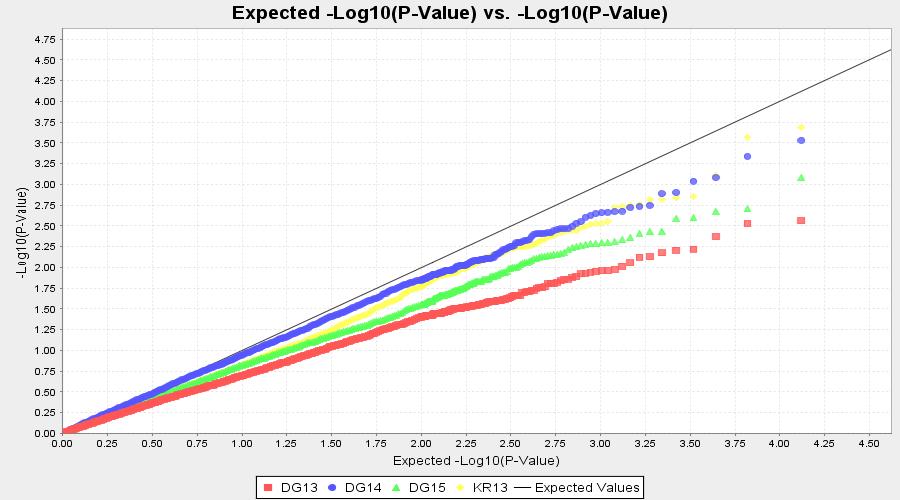


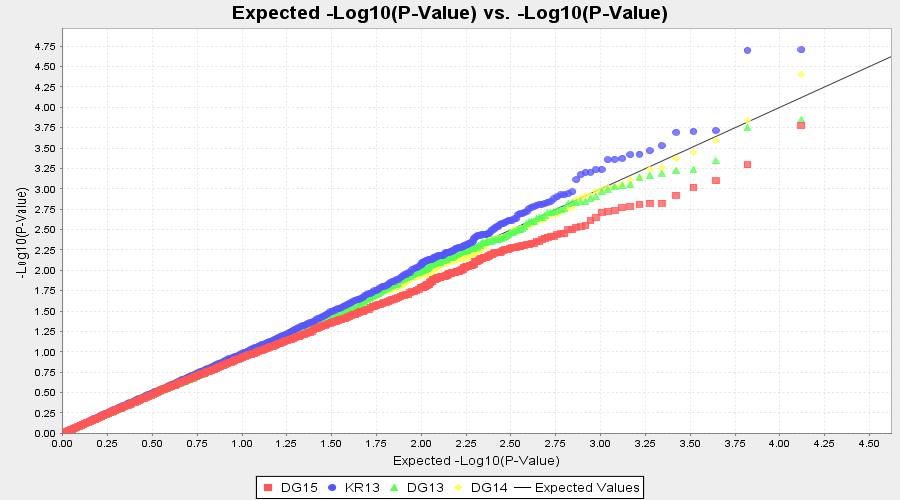


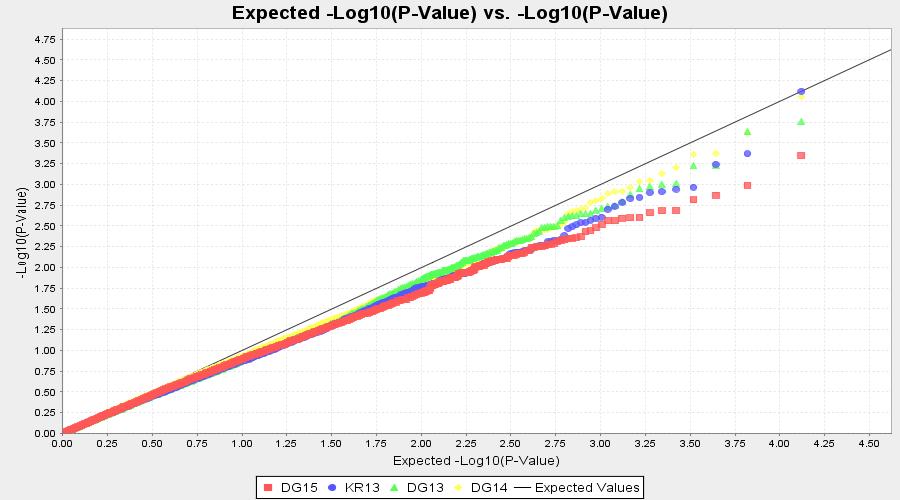


Sup. Figure 3. QQ plots for SRT marker-trait association analysis using the SNPs markers set and the following four models: GLM + Q, GLM +PCA, MLM (K+Q Model) and MLM (PCA+K Model). The black line is the expected line under the null distribution.


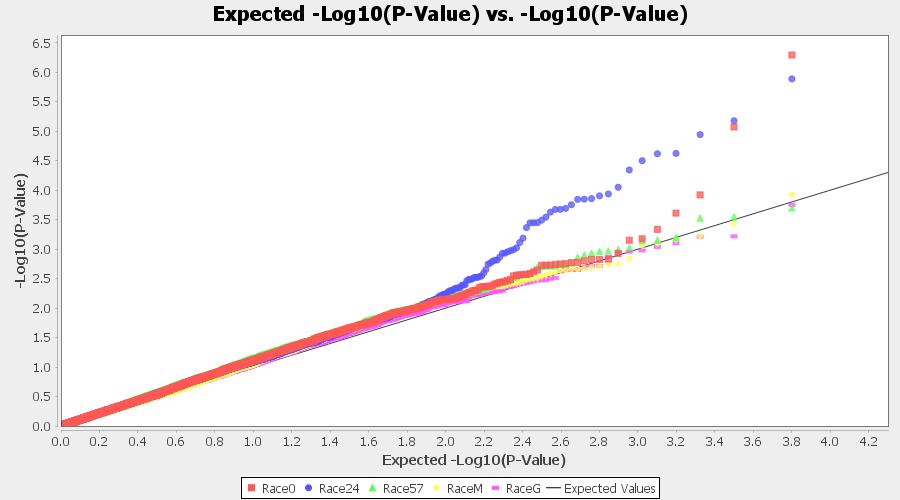


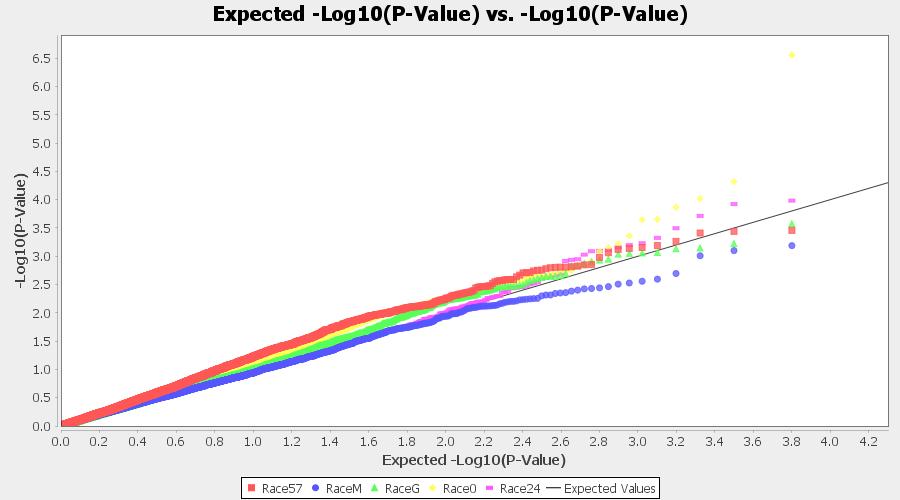


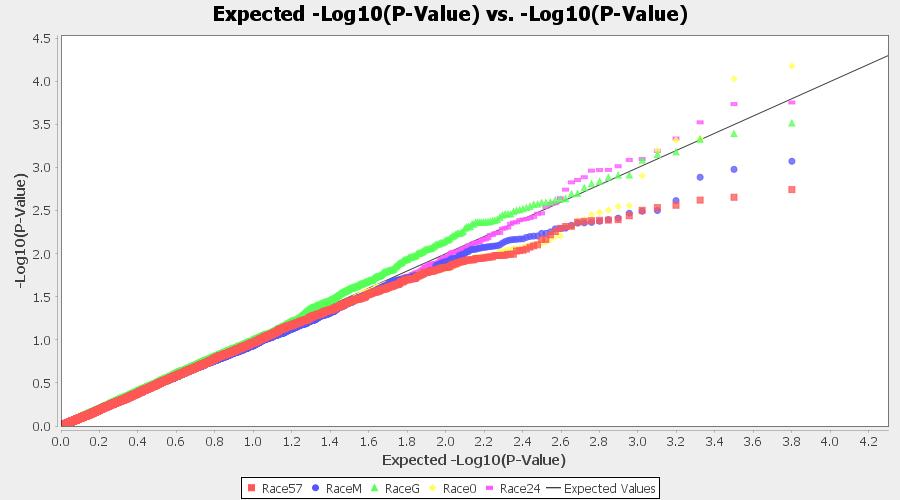


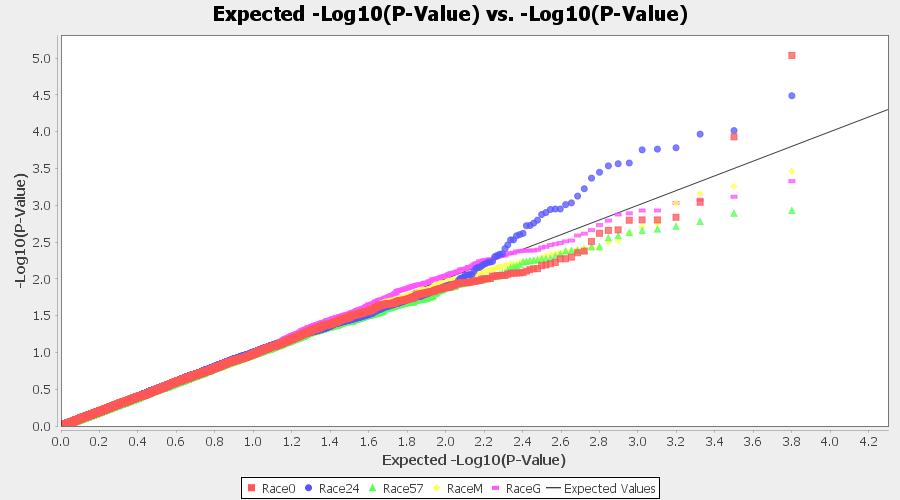


Sup. Figure 4. QQ plots for APS marker-trait association analysis using the SNPs markers set and the following four models: GLM + Q, GLM +PCA, MLM (K+Q Model) and MLM (PCA+K Model). The black line is the expected line under the null distribution.


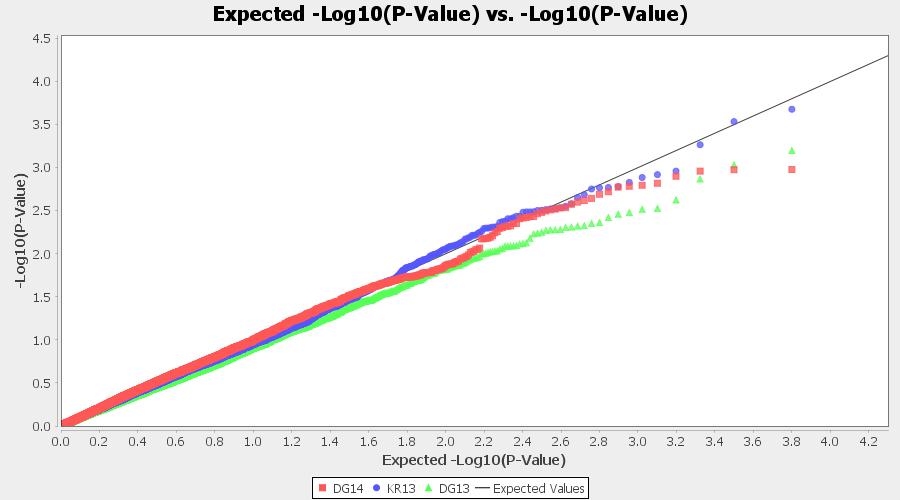


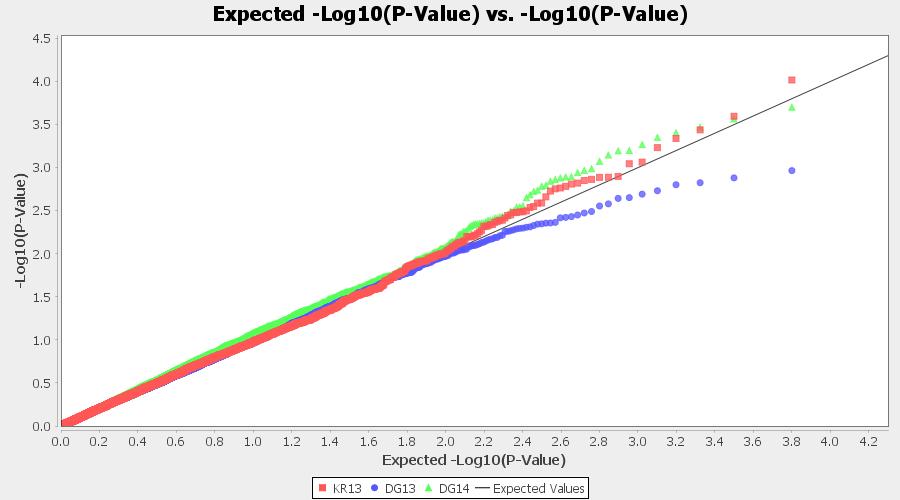


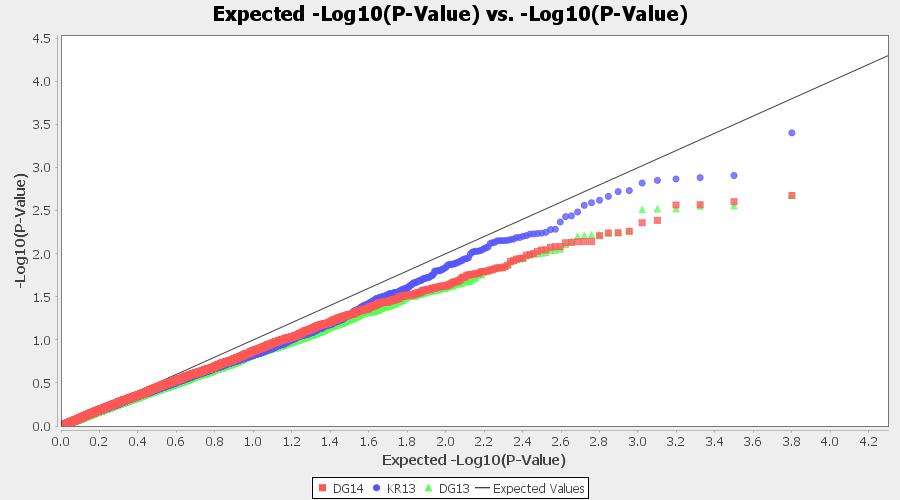


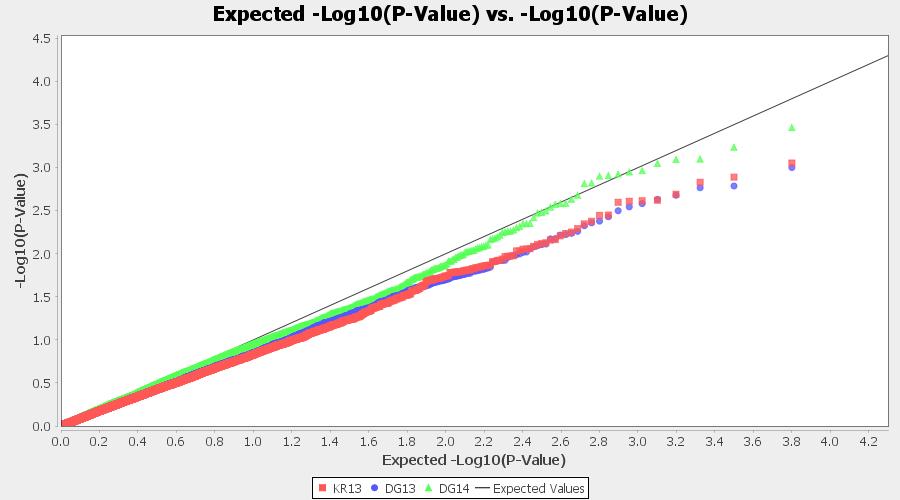

Supplement: Supplementary file 1 [file Data_Sheet_1.DOCX]
